# Dynamic Pore-scale Reservoir-condition Imaging of Reaction in Carbonates Using Synchrotron Fast Tomography

**DOI:** 10.3791/53763

**Published:** 2017-02-21

**Authors:** Hannah P. Menke, Matthew G. Andrew, Joan Vila-Comamala, Christoph Rau, Martin J. Blunt, Branko Bijeljic

**Affiliations:** ^1^Department of Earth Science and Engineering, Imperial College London; ^2^Carl Zeiss X-Ray Microscopy; ^3^Diamond Manchester Imaging Branchline (I13-2), Diamond Lightsource

**Keywords:** Engineering, Issue 120, Carbon Capture and Storage, Acid Injection, x-ray tomography, Synchrotron Pink Beam, Reservoir Condition, Carbonate Dissolution

## Abstract

Underground storage permanence is a major concern for carbon capture and storage. Pumping CO_2_ into carbonate reservoirs has the potential to dissolve geologic seals and allow CO_2_ to escape. However, the dissolution processes at reservoir conditions are poorly understood. Thus, time-resolved experiments are needed to observe and predict the nature and rate of dissolution at the pore scale. Synchrotron fast tomography is a method of taking high-resolution time-resolved images of complex pore structures much more quickly than traditional µ-CT. The Diamond Lightsource Pink Beam was used to dynamically image dissolution of limestone in the presence of CO_2_-saturated brine at reservoir conditions. 100 scans were taken at a 6.1 µm resolution over a period of 2 hours. The images were segmented and the porosity and permeability were measured using image analysis and network extraction. Porosity increased uniformly along the length of the sample; however, the rate of increase of both porosity and permeability slowed at later times.

**Figure:**
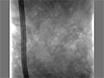


## Introduction

A major concern of carbon capture and storage (CCS) is long-term storage security^1^,^2^. Carbon dioxide, CO_2_, injected into the subsurface will dissolve in the host brine and form carbonic acid^3^,^4^,^5^. This acidic brine has the potential to react with and dissolve the surrounding rock, particularly if the host rock is limestone^6^. Dissolution can be favorable and allow for continued formation permeability^7^ and greater storage permanence^8^. However, geologic seal integrity may be compromised by this dissolution and allow CO_2_ to migrate to the surface^9^. Accurate predictive modeling of storage permanence is thus dependent on fully understanding dissolution in the brine-rock system and the distribution and the rate of fluid movement in the subsurface^10^,^11^,^12^.

However, the nature and the rate of dissolution in carbonates is dependent on both the properties of the brine^13^,^14^,^15^,^16^ and the host rock^17^. The dissolution rates are also strongly dependent on brine temperature and pressure^6^, making the development of experimental techniques for measuring complex time-dependent processes at representative reservoir conditions vital.

Previous experiments have observed that field-scale reaction rates are typically orders of magnitude lower than experimental batch reactor measurements^18^,^19^. Weathering, mineral heterogeneity, and incomplete mixing in a heterogeneous flow field are possible explanations for this phenomenon. However, it is not possible to assess the most significant factors without direct observation of the evolving pore space during reaction. Thus, dynamic pore-scale experiments are required to provide both the insights into the interplay between transport and reaction and to validate predictive models.

An established experimental method for studying pore-scale processes in carbon storage applications is X-ray microtomography (µCT)^20^,^21^. µ-CT has several benefits: it achieves high spatial resolutions of down to around 1 µm, it is non-invasive, and provides three-dimensional images. Limestone dissolution has been studied at the core (~cm) scale^22^ and it was found that rock-brine reaction increases physical heterogeneity. To advance understanding of how different transport and reaction conditions alter the complex solid and pore structures it is necessary to measure reaction-induced changes in pore-space geometry, topology and flow in subsurface rock systems at reservoir temperatures and pressures and at a higher resolution, to investigating in detail pore-scale processes. This paper describes a method of studying reactive dissolution processes in rock with complex pore structures and focus on measuring the time and spatially dependent reaction rate between a CO_2_-acidified brine and limestone rock at reservoir conditions.

There have been several studies that have looked at reaction in complex carbonates^23^,^24^,^25^,^26^,^27^, but due to experimental or imaging constraints they have been either limited to pre and post reaction images or were not completed at representative subsurface conditions. Menke *et al.*^28^ has performed dynamic *in situ* imaging of reaction between a CO_2_-acidified brine and Ketton limestone at the pore scale over a period of several hours and at a temperature and pressure representative of an aquifer at approximately 1 km in depth. However, Ketton is a relatively homogenous rock with large grains that is easy to image in very little time (~17 min) and with few projections (~400). Most carbonate rocks have complex pore structures that require many projections to accurately resolve which can be a very time intensive process using traditional µ-CT — either with a monochromatic beam at a synchrotron source or with bench-top X-ray scanners. Thus, a fast method of tomography is needed to see reaction-induced changes in heterogeneous carbonates dynamically.

The amount of time it takes to image a sample is controlled by the flux of the X-ray source. One method of scanning quickly is to use the polychromatic beam of a synchrotron source^20^. This so-called 'Pink Beam' provides orders of magnitude more intense light than bench-top sources and therefore images can be taken on the tens-of-second rather than hour time scales. An undulator that consists of a periodic structure of dipole magnets produces the Pink Beam. The electron beam is forced to undergo oscillations as it traverses the magnets and as a consequence radiates energy. The energy produced is concentrated to narrow wavelength bands and is very intense. Mirrors and filters are then used to narrow the spectrum of light to suit experimental needs. Mirrors absorb the high-energy spectrum while filters absorb the lower energies. It is therefore possible to narrow the spectrum to the desired band of radiation using only these tools.

However, using this intense X-ray flux is not without its challenges. The lower energy X-rays of the Pink Beam spectrum are absorbed by the sample as heat. This can interfere with the temperature control of the *in situ* apparatus and cause CO_2_ to exsolve from solution^20^. CO_2_-saturated brine is very sensitive to both heat and pressure and therefore a small change in thermal equilibrium can significantly change the pH of the *in situ* fluid^5^. Thus, careful design and control elements for the X-ray spectrum must be incorporated into the beam line equipment prior to imaging.

Fast tomography also produces a vast amount of data at a high rate. The limitations of data read out from the camera and subsequent storage provide a substantial technological challenge. Some have overcome this by taking several consecutive scans and storing them on the camera memory before reading them to external data servers. However, this requires that the experiment be relatively short as the camera memory can only hold a finite volume of data. Binning the data on the camera also reduces transfer time as it reduces the volume of data needing to be transferred, but it has the potential to reduce the quality of the images. Alternatively, the data can be transferred off the camera after each scan before starting the next, which will increase the total time between scans. This study used the latter method with each image acquisition taking ~45 seconds and data read off taking an additional ~30 s.

When taking scans at a high rate, the sample stage must spin much faster than with traditional scanning and therefore the potential angular stress on the core holder is great. Carbon fiber, while X-ray transparent, is flexible when stressed. If the sample moves during image acquisition image blurring can occur. The core holder sleeve was designed to be as short as possible to mitigate these potential stresses. Additionally, flexible polyether ether ketone (PEEK) tubing was used on all elements of the experimental apparatus close to the stage so that the stage was free to rotate. One drawback of using PEEK tubes is that it is permeable to CO_2_ on diffusive timescales. Fluid residing in the lines for long periods will gradually become desaturated over a period of about 24 h. All lines that were not near the core holder were made of stainless steel and the fluid was pre-equilibrated in a vigorously mixed Hastelloy reactor heated and pressurized to experimental conditions^23^,^29^,^30^.

The experimental apparatus is depicted in **Figure 1**. Reservoir temperature is maintained in the core holder by wrapping the exterior of the sleeve in an X-ray transparent heating tape and inserting a thermocouple through the radial port of the cell and into the confining fluid. A Proportional Integral Derivative (PID) controller then regulated temperature to within 1°C. Pressure and flow conditions were maintained using three high-pressure syringe pumps that are accurate to a flow rate of 0.001 mL/min. Two salts were used for the experiment, a highly absorbing 25% wt KI unreactive brine and a low absorbing 1% wt KCl, 5% wt NaCl reactive brine. The difference in attenuation made it easy to see the arrival of reactive brine in the core making dead volume calculations unnecessary.

## Protocol

### 1. Imaging Strategy Design

1. Calculate the X-ray spectra of the beamline at the highest pink beam energy and flux in order to predict imaging performance using the experimental tuning curve and measuring filter transmissions. An example of the X-ray spectra of the Diamond Lightsource I13-2 'Pink Beam' is shown in **Figure 2**.
2. As the lower energy X-rays cause heating of the sample and do not add to imaging contrast, filter out the lower part of the X-ray spectrum such that only the highest energy X-rays are used in imaging the sample. Choose in line filters of materials that absorb at the desired low wavelengths of light suitable to the light source by calculating the theoretical filter transmission at the available light wavelengths^31^,^32^. Here, use aluminum and gold for the beam line at this light source.
  1. Use a band-pass filter consisting of a combination of X-ray filters as high-pass and an X-ray mirror operating near the critical angle as low-pass filter. In this case, use a set of 0.2 mm pyrolytic carbon and 0.2 mm aluminum filters and for the mirror a platinum-coated strip under an incident angle of 1.15 mrad. The mirror reflects only light below 30 keV and additional inline filters have been installed of 2 mm Al and 0.1 µm Au which have absorption peaks at 13 and 22 keV respectively to filter more of the lower energy X-rays. **Figure 3** depicts the beamline imaging apparatus.
3. Choose a scintillator that scintillates abundantly at the beamlines available light frequencies and flux. In this case, the scintillation screen is made of 250 µm-thick cadmium tungstenate (CdWO_4_), which is stacked with 750 µm-thick lead tungstenate (PbWO_4_). Then choose an objective lens and camera that have an appropriate field of view and snapping time resolution for the experimental requirements. In this case, couple a 1.25X objective lens with an aperture of 0.04 with a PCO EDGE 5.5 CMOS camera and used to capture a 4-mm field of view with a frame rate of 0.001 s.
4. Choose the 'flyscan' technique for image acquisition as this method of stage rotation reduces sample vibration. Traditional acquisition requires that the stage stop at each angular increment, take a projection, and then move to the next angle. The image acquisition during these dynamic tomographies was done with a 'flyscan', which takes tomographic scan as the stage is moving and assumes an angular increment such that the difference between each successive projection is small. The 'flyscan' method eliminates the small vibrational effects of the start-and-stop motion and provides higher quality image more quickly.

### 2. Assembly of Equipment and Cell

1. **Load the core into the cell in preparation for core flooding.**
  1. First, wrap the core in one layer of aluminum foil and insert into a sleeve (*e.g.*, Viton) (**Figure 4**).
  2. Cut the sleeve to size so that it is 2 mm shorter than the combined length of core and interior end fittings. The end fittings are 1/16" national pipe thread (NPT) to union fittings that have been machined to 5 mm in exterior diameter, while the sleeve is 4 mm in interior diameter.
  3. Stretch the sleeve over the 5 mm end fittings to create a tight seal. Ensure that there is not any space between the end fittings and the core to ensure that the confining pressure does not over compress the sleeve and pinch off flow.
  4. Wrap the fittings and sleeve in two additional layers of aluminum both to prevent gaseous CO_2_ from diffusing into the confining fluid and to keep the sleeve in place on the fittings and prevent a hydraulic pathway from connecting the confining and pore fluids.
  5. Put the core holder back together by sliding the tubing and seals back in place and seal the end caps and end fittings by replacing the bolts.
2. Mount the core holder on the stage and connect the flow and the electrical lines.
3. Test the stage rotation and ensure that all flow and electrical lines are free to rotate from -90° to 90°.
4. **Take a dry scan of the entire core prior to beginning the experiment.**
  1. Scan the core in overlapping sections around 4 mm in width and length. Calibrate the scan exposure time to an average count value of around 15,000, which ensures a high signal to noise ratio without over-saturating the scintillator. Take each dry scan with at least 2,400 projections to maintain phase contrast and edge sharpness.
  2. Take flat and dark images of the scintillator so that any damage and external noise can be accounted for during reconstruction. Take flats by moving the core holder out of the field of view and taking an image of just the scintillator with the beam on. Take darks using the same method with the beam off.

### 3. System Pressurization

1. **Load a 1% wt potassium chloride (KCl) 5% wt sodium chloride (NaCl) brine into the disassembled reactor by pouring fluid in through the top of the reactor vessel.**
  1. Add powdered carbonate rock to achieve the desired brine acidity. In this case no carbonate was added.
  2. Reassemble the reactor by tightening the bolts and rewrapping it with heating tape and inserting the temperature probe into the top.
  3. Load CO_2_ into the injection pump by opening valve 1 (V1 in **Figure 1**).
  4. Close Valve 1 and pressurize the injection pump to 100 bar.
  5. Open Valve 2 to flood the reactor with CO_2_. Heat the reactor to 50 °C using a PID controlled heating wrap in combination with a temperature probe and continuously stir with an entrainment stirrer driven by an external electric motor. Equilibrate the brine with CO_2_ at 10 MPa and 50 °C for between 2 and 6 h to ensure that the brine is completely saturated with CO_2_ and the carbonate is fully dissolved.
2. **Prior to connecting the core holder, completely purge the system of air and possible precipitants in the lines from previous experiments. To do this, connect the lines above and below the core holder to bypass the core holder (U1 and U2).**
  1. Load deionized (DI) water into the receiving pump through valve 11 by setting the receiving pump to refill.
  2. Open valves 7, 4, and 3 and use the receiving pump on constant pressure mode to drive DI water backwards through the system and out of valve 3 below the reactor. Use approximately ten system volumes to ensure the lines are clear of air and rinsed clean.
3. **Empty the receiving pump and then load a brine of 25% wt KI into the receiving pump through valve 11 and load DI water into the confining pump through valve 10.**
  1. Close valve 10 and open valves 8 and 6. Use the confining pump to confine the core at 2 MPa.
  2. Close Valve 11 and pressurize the receiving pump to 10 Bar.
  3. Open valves 9, 7, 4, & 3 and use the resulting pressure drop to drive KI doped brine through the core.
  4. Step up the confining and pore pressures incrementally until a reasonable flow rate is established. Drive approximately two complete system volumes of brine through the core and drain the fluid through valve 3 below the reactor. In this way all air is purged from the system and the core is flooded with high contrast brine that makes the arrival of undoped reactive brine easy to observe.
  5. Close valve 3 and incrementally increase the confining and pore pressures until the core is confined at 12 MPa and the pore pressure is 10 MPa. Switch on the PID controller to bring the core to 50°C.
  6. Stop the receiving pump, close valve 3, and open valve 5 at the base of the reactor to connect the reactor system to the core.

### 4. Fluid Flow and Image Acquisition

1. Center the middle of the core in the field of view and take 2-D projections continuously as the core is flooded to track the core flooding progress. Take 2-D projections by turning on the pink beam and using the camera to take images without rotating the stage. Starting 2-D projections before reactive fluid injection gives a clear before brine image that will later be compared to subsequent brine filled images.
2. **Set the receiving pump to refill at the desired flow rate thus pulling fluid from the reactor through the core at the desired flow conditions while leaving the injection pump to regulate pressure from the front end.**
  1. Monitor the 2-D projections for changes in attenuation that signal the arrival of reactive brine. When reactive brine arrives, the transmission of the core will increase and the 2-D projections will brighten considerably as more light hits the scintillator and the doped brine is displaced by the highly X-ray transparent reactive fluid. If there is no attenuation difference between reactive and non-reactive brine then, depending on the beam line spectrum, restarting the experiment from step 2.1 with a higher salt concentration KI or using a different highly absorbing salt may be required.
  2. Stop the 2-D scans and 3-D take tomographies successively as fast as the imaging apparatus allows. Use approximately 1,000 projections per scan. Scan the core using only 180° of rotation (as opposed to the traditional 360°). While using fewer degrees of rotation decreases the signal to noise ratio, it is faster and helps to avoid stretching and tangling the flow and electrical lines. Take 3-D scans until either the time limit is reached or the core looks sufficiently dissolved that there is an imminent danger of internal structural collapse (and thus causing the loss of both the confining pressure and future whole core dry scan data).
3. **After the last scan is taken, depressurize the system efficiently to avoid reacting the core any further.**
  1. First stop the receiving pump. Then close valve 5 connecting the reactor to the rest of the system.
  2. Step the system pressure down using the confining and receiving pumps keeping around 1MPa more pressure on the confining fluid.
  3. Once within 1 MPa of atmospheric pressure is reached, open the confining and receiving pumps using valves 10 and 11 and run in constant flow mode to drain any remaining fluid.
  4. Shut off the PID controller and open the 4-way union (U2) at the top of the core holder to release any remaining system pressure.
  5. Slowly loosen the confining line while catching excess DI confining water with absorbent paper. Close valves 6 and 7 and disconnect union 1 and the electric lines.
  6. Loosen the stage clamp and remove the core holder from the stage.
4. Carefully remove the core assembly from the core holder and then disconnect the sleeve from the interior end fittings. Do not remove the core from the sleeve because doing so may damage the fragile reacted core. Place the sleeve-covered core in a beaker full of DI water to dilute any potentially reactive brine and stop all reaction.
5. Dry the entire core in a 60 °C oven for at least 12 h. Then remount the core on the stage using a traditional sample mount, and scan it again at the same resolution and projections as the initial dry scan.

### 5. Image Processing

1. Correct the reconstructed images for any beam hardening associated with using a polychromatic beam by assuming that any affects are radially symmetric Gaussian functions^33^.
2. Filter the images using an edge-preserving filter such as non-localized means to increase signal to noise^34^,^35^ (see Supplemental File).
3. **Segment the dry scan images using a watershed segmentation**^36^** algorithm and define the seeds as rock and void(see**Supplemental File**)**.
  1. Take the first image of the core with reactive brine and register each subsequent image to the first image and resample it using the Lanczos^37^ resampling method with the first image as a reference. As ongoing reaction tends to blur edges, watershed segmentation on the images is not sufficient for accurate segmentation.
  2. Subtract each reacted core image from the first image to get the difference image. Segment the difference images into change and no change. Register the segmented dry scan to the first reactive scan and then subtract the segmented change from the segmented dry scan to achieve the segmented reacted images^38^.

### 6. Modeling

1. Use the binarized images as inputs into a either a direct Navier-Stokes flow solver^39^,^40^, or a network extraction model^41^ (**Figure 8**) to characterize permeability changes and provide physical insights into the dynamics of dissolution.

## Representative Results

The reaction was imaged between calcite and unbuffered scCO_2_ saturated brine in 4 mm-diameter 1.2 cm-long Portland carbonate core^42^. Portland carbonate is a relatively pure (<99%) calcite oolite with a complex heterogeneous pore structure^43^. The low energy X-rays were filtered by passing the beam through 2 mm of Al and 0.1 µm of Au. A CdWO_4_ scintillator with a 1.25X objective lens and a PCO EDGE camera were used in the detector assembly. The dry scans were acquired with 4,000 projections while the dynamic scans had 1,000 projections each. Total acquisition time was ~1 minute 15 seconds per scan with ~100 scans taken over a period of 2 hours.

Reconstruction and artifact removal was completed using the Diamond Lightsource proprietary software. Each image consists of 2000^3^ voxels, which were then binned to increase signal to noise resulting in an image of 1000^3^ voxels at a resolution of 6.1 µm (**Figure 5**). The images were then processed using the image processing modules in Avizo 8.1 and ImageJ programs (see Supplemental File). Each image required approximately 12 CPU hours and 3 GPU hours of processing on a computer with a 3.0 GHz CPU and a Tesla K20C GPU.

The segmented images were analyzed as a time series for porosity changes by counting the number of voxels of pore and rock. During dissolution porosity increases with time (**Figure 6**). Visual inspection of the segmented images (**Figure 7**) shows the presence of a channel in the direction of flow. When porosity is plotted as a function of both time and distance from the sample inlet it is clear that a channel is formed in the first hour and then widened as the experiment continues (**Figure 8**).

The segmented images were then used as input into a network extraction model to analyze permeability changes (**Figure 9**). It was found that there was a sharp increase in permeability during the initial hour, but then the permeability stabilized at later times.


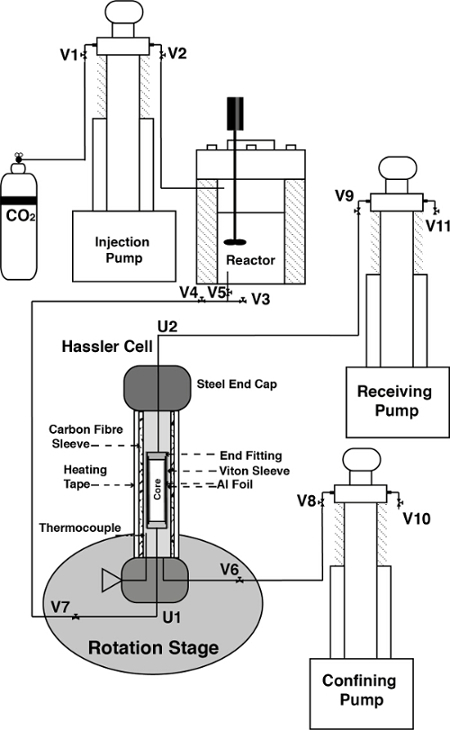
**Figure 1. The *in situ* experimental apparatus.** CO_2_ is pressurized by the injection pump and used to equilibrate brine in the reactor. Reactive brine is pulled through core assembly by the receiving pump. The cell is confined by DI water in the confining pump and heated using heating tape controlled by a thermocouple in the confining fluid. The experimental system is connected together using tubing and fluid flow is directed using Valves (V) and Unions (U). Please click here to view a larger version of this figure.


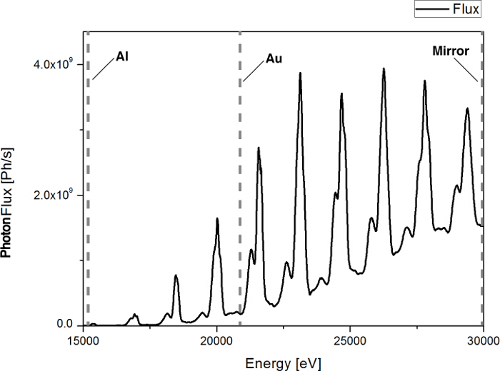
**Figure 2. The X-ray spectra of the Diamond Lightsource I-13I pink beam calculated using both the experimental tuning curve and theoretical mirror reflectivity and filter transmission.** Mirrors absorb energies above 30 keV; Al and Au filters absorb energies below 13 and 22 keV respectively. Please click here to view a larger version of this figure.


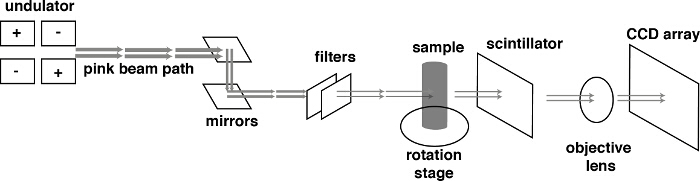
**Figure 3. The beam line imaging apparatus.** The Al and Au sheets filter the pink beam and the remaining X-rays hit the core assembly. A portion of the X-rays are absorbed by the sample while the rest pass through the sample and hit the scintillator which fluoresces in the visible spectrum. This visible light is then focused by the objective onto the CCD, which translates that light into a pixelated digital image where the pixel intensity value is a function of the number of X-rays that are absorbed by the scintillator. Please click here to view a larger version of this figure.


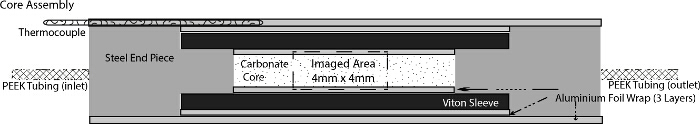
**Figure 4. The core assembly inside the core holder.** PEEK tubing is attached to the interior end fittings and threaded through the steel end caps. The core is wrapped in aluminum foil and inserted into the sleeve. The sleeve is then stretched over the end fittings to create a watertight seal and two additional layers of aluminum foil are added to hold everything in place and prevent gas diffusion. The thermocouple is secured to the outside of the core assembly with the outer layer of adhesive aluminum foil. Figure modified from Menke *et al.*^42^. Please click here to view a larger version of this figure.


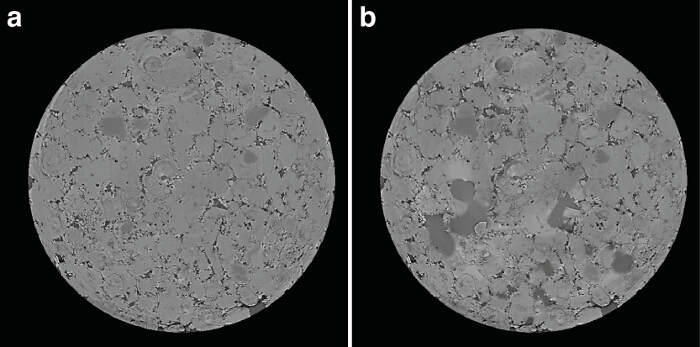
**Figure 5. A 2-D slice of the reconstructed image before (a) and after (b) dissolution. **The lighter areas are grain and the darker areas are pore. Blurring at the edges of the grain/pore boundary can be seen in the reacted portion of the pore space (b). Please click here to view a larger version of this figure.


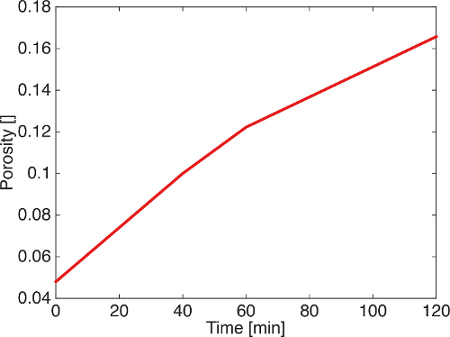
**Figure 6. Porosity plotted with time. **Porosity increases linearly with a small decrease in slope in the second hour of dissolution. Figure modified from Menke *et al.*^42^. Please click here to view a larger version of this figure.


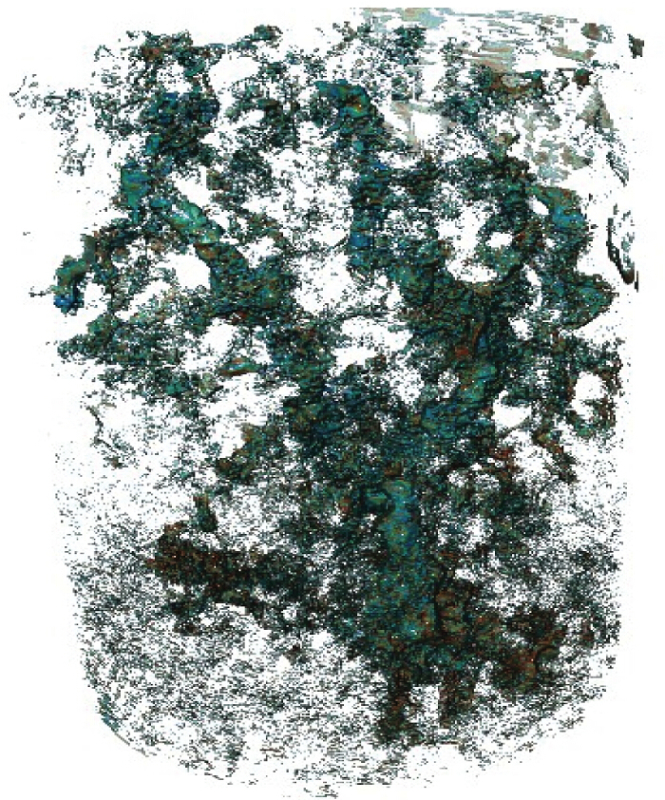
**Figure 7. A 3-D rendering of the change in porosity at 60 minutes into the experiment, where green represents the greatest change in porosity and red the least. **A clear porous channel created by fluid-solid chemical reaction is seen in the center of the core where dissolution is greatest. Figure modified from Menke *et al.*^42^. Please click here to view a larger version of this figure.


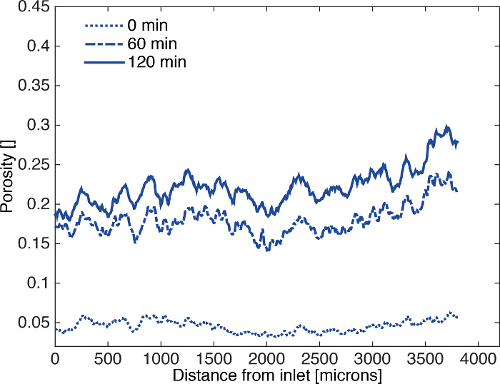
**Figure 8. Profiles of porosity as a function of distance from the sample inlet. **Porosity is uniform along the axis of dissolution, but the rate of dissolution changes as a function of time. Figure modified from Menke *et al.*^42^. Please click here to view a larger version of this figure.


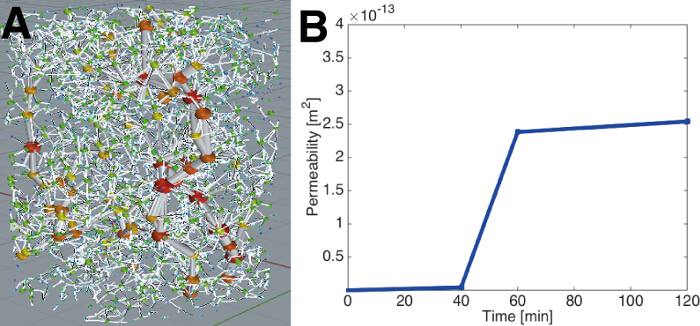
**Figure 9.** (**A**) A network extraction performed on the segmented images is shown at 60 minutes, showing large pore spaces (balls) and their connections (tubes). (**B**) The computed permeability is shown to increase with time with a sharp rise between 40 and 60 minutes as a wide dissolution channel is established. Figure modified from Menke *et al.*^42^. Please click here to view a larger version of this figure.

## Discussion

The most critical steps for dynamic imaging of reaction in heterogeneous pore structures at reservoir conditions are: 1) accurate temperature control of the cell inside the pink beam; 2) successful core holder stability on a fast moving stage; 3) efficient data processing and storage techniques; and 4) effective segmentation of time-resolved images.

Temperature control is essential for reservoir condition imaging using a Pink Beam. If the temperature rises above the reactor temperature, CO_2_ will exsolve in the pore space and both change the pH of the brine and create ganglia of supercritical CO_2_ in the pore space that could change the nature of dissolution^44^. The use of filters to absorb the lower energy X-rays is critical for removing this additional temperature stress which allows the thermocouple and heating wrap to effectively control temperature externally. However, filters lower the total energy throughput of the beam and thus must be used sparingly so as not to significantly increase total acquisition time. Moreover, filter type and thickness must be tailored to the specific energy wavelengths and throughput of the beam line.

The core holder undergoes rotational and vibration stresses during tomography acquisition that can cause the carbon fiber sleeve to shake during stage rotation and blur the projections. To minimize this potential, the core holder is designed as a short 6 cm sleeve for use at synchrotrons. This sleeve would not be favorable for use with bench top scanners, as the steel end fittings would inhibit minimization of source-sample distance and geometric magnification. However, with a parallel light source these are not concerns.

Each tomographic scan taken in a series can have a size of over 20 GB meaning that a series of 100 scans will be 2 TB in size. When taking many scans in a row very quickly both the instrument bandwidth and storage options provide substantial data management challenges. The experimental imaging apparatus must be designed with these constraints in mind so as to fully realize the dynamic imaging potential of fast tomography. Data transfer bottlenecks must be identified before starting the experiment and the technology infrastructure adapted so that issues such as camera read off speed, transfer bandwidth, and storage write speed do not inhibit acquisition speed potential.

Effective segmentation of the time-resolved images of dissolution provides a challenge. When a tomographic scan is taken in a changing system the edges of the solid-liquid boundary can become blurred. This blurring makes traditional segmentation techniques such as watershed, which works on the assumption that the boundaries will be the regions with the highest attenuation gradient, much less successful. To circumvent this, the difference image of the unreacted and reacted images is calculated which provides an image of only regions of change. This method allows for successful segmentation of the continuously changing pore structure.

Synchrotron fast tomography coupled with a reservoir scale apparatus is a powerful experimental method that can be adapted to explore a range of applications including multiphase flow processes, advection-dispersion, and transport in chemically heterogeneous mediums. However, the current apparatus is limited to a time resolution on the order of seconds, single-phase experiments, and small sample sizes. Future design upgrades may include additional pumps for three-phase capabilities, increasing flux to be able to penetrate larger mediums, better reconstruction techniques that allow for fewer projections to be taken per scan, and multivariate approaches to image acquisition and segmentation that can further improve information depth, breadth, and accuracy.

## Disclosures

The authors have nothing to disclose.
